# *flhDC*, but not *fleQ*, regulates flagella biogenesis in *Azotobacter vinelandii*, and is under AlgU and CydR negative control

**DOI:** 10.1099/mic.0.2008/017665-0

**Published:** 2008-06

**Authors:** Renato León, Guadalupe Espín

**Affiliations:** Departamento de Microbiología Molecular, Instituto de Biotecnología, Universidad Nacional Autónoma de México, Apdo Postal 510-3, Cuernavaca, Morelos 62250, Mexico

## Abstract

*Azotobacter vinelandii* is a nitrogen-fixing soil bacterium that undergoes differentiation to form cysts resistant to desiccation. Upon encystment, this bacterium becomes non-motile. As in enteric bacteria, motility in *A. vinelandii* occurs through the use of peritrichous flagella. *Pseudomonas aeruginosa*, a phylogenetically close relative of *A. vinelandii*, possesses a single polar flagellum. The FlhDC proteins are the master regulators of flagella and motility in enterobacteria, whereas FleQ is the master regulator in *P. aeruginosa*, and it is under AlgU (sigmaE) negative control. At present, nothing is known about the organization and expression of flagella genes in *A. vinelandii*. Here, we identified the flagella gene cluster of this bacterium. Homologues of the master regulatory genes *flhDC* and *fleQ* are present in *A. vinelandii*. Inactivation of *flhDC*, but not *fleQ*, impaired flagella biogenesis and motility. We present evidence indicating that a negative effect of the AlgU sigma factor on *flhDC* expression causes loss of motility in *A. vinelandii*, and that CydR (a homologue of Fnr) is under AlgU control and has a negative effect on *flhDC* expression. Taken together, these results suggest the existence of a cascade consisting of AlgU and CydR that negatively controls expression of *flhDC*; the results also suggest that the block in flagella synthesis under encystment conditions centres on *flhDC* repression by the AlgU–CydR cascade.

## INTRODUCTION

*Azotobacter vinelandii*, a Gram-negative bacterium belonging to the *Gammaproteobacteria*, is closely related to bacteria of the genus *Pseudomonas* (Rediers *et al.*, 2004), and undergoes a differentiation process to form cysts resistant to desiccation. In *A. vinelandii*, motility occurs through the use of peritrichous flagella. When induced for encystment, this bacterium becomes non-motile (Sadoff, 1975). To the best of our knowledge, studies on the genes involved in motility in this organism have not been carried out. Genes involved in the biogenesis and function of flagella have been extensively studied in *Escherichia coli* and *Salmonella*, where motility also occurs by peritrichous flagella. In those bacteria, flagella and motility genes comprise a large and complex regulon, with more than 50 genes organized in at least 17 operons (Macnab, 1996). The flagella operons are classified into three hierarchical transcriptional classes, where the class 1 *flhDC* operon is the master regulator of flagella and motility (for a review see Aldridge & Hughes, 2002). FlhDC proteins are activators of the class 2 genes, which include those encoding proteins involved in the formation of the hook basal body, the flagella sigma factor FliA, and its anti-sigma factor FlgM. FliA is necessary to activate transcription of the class 3 genes, and these include *fliC*, which encodes the structural component of the filament, the flagellin. Transcription of *flhDC* is initiated at six start sites, and its regulation is very complex (Clarke & Sperandio, 2005; Francez-Charlot *et al.*, 2003; Kutsukake, 1997; Soutourina *et al.*, 1999; Wei *et al.*, 2001; Yanagihara *et al.*, 1999).

In *Pseudomonas aeruginosa*, motility occurs through the use of a single polar flagellum. Flagella genes are clustered in three regions of the chromosome. Transcription of the flagella genes requires a number of regulatory proteins, including FleQ (Arora *et al.*, 1997) and the two-component system FleSR (Ritchings *et al.*, 1995), acting in a four-tiered transcriptional regulatory circuit (Dasgupta *et al.*, 2003). The master regulator FleQ, an NtrC-like transcriptional activator, belongs to the top tier of the flagella hierarchy, and is required to activate all other flagella genes, with the exception of *fliA* (Arora *et al.*, 1997; Dasgupta *et al.*, 2003; Jyot *et al.*, 2002). The anti-activator protein FleN negatively controls the activity of FleQ (Dasgupta & Ramphal, 2001).

In *P. aeruginosa* and *A. vinelandii*, the alternative sigma factor AlgU (also named AlgT) controls alginate biosynthesis. The mechanism by which AlgU exerts this control is well understood (Martínez-Salazar *et al.*, 1996; Núñez *et al.*, 2000; Ramsey & Wosniak, 2005). The anti-sigma proteins MucA and MucB negatively control AlgU activity (Mathee *et al.*, 1997; Schurr *et al.*, 1996; Xie *et al.*, 1996). External stresses affecting the folding of periplasmic proteins initiate the cleavage of MucA, and the release of AlgU (Qiu *et al.*, 2007). In *P. aeruginosa*, this sigma factor is required to activate the alginate biosynthesis operon (Martin *et al.*, 1993; Wozniak & Ohman, 1994). In *A. vinelandii*, AlgU is required for activation of the alginate biosynthesis genes *algD* and *algC* (Campos *et al.*, 1996; Gaona *et al.*, 2004). Thus, in both *P. aeruginosa* and *A. vinelandii,* mutations inactivating *algU* abrogate alginate synthesis (Moreno *et al.*, 1998), whereas mutations in *mucA* produce an alginate-overproducing phenotype (Martin *et al.*, 1993; Núñez *et al.*, 2000).

In *P. aeruginosa*, AlgU activity has a negative effect on flagellum synthesis (Garrett *et al.*, 1999). Tart *et al.* (2005) showed that the flagella regulon is significantly downregulated in the presence of AlgU, and that increased expression of *fleQ* reverses the AlgU-mediated inhibition, and thus they concluded that AlgU inhibits expression of FleQ. The mechanism of this inhibition has been shown to be indirect, and it acts by promoting the expression of the transcriptional regulator AmrZ (AlgZ), which interacts directly with the *fleQ* promoter as a repressor (Tart *et al.*, 2006).

When vegetative motile cells of *A. vinelandii* are induced for encystment, they lose motility (Sadoff, 1975). We show here that this loss is caused by the activity of the sigma factor AlgU. Thus, similar to the findings for *P. aeruginosa*, we found a negative effect of AlgU on motility and flagella synthesis.

The organization and expression of flagella genes in *A. vinelandii* are unknown. A search of the *A. vinelandii* genome for the flagella regulon was carried out in this study, and revealed the presence of homologues of *flhDC* and *fleQ,* which are the master regulators in *E. coli* and *Pseudomonas*, respectively. Inactivation of these genes indicated that *flhDC*, but not *fleQ*, is required for flagella biogenesis and motility in *A. vinelandii*. We also show that AlgU and CydR have a negative effect on *flhDC* expression.

## METHODS

### Microbiological procedures.

The bacterial strains and plasmids used in this work are shown in Table 1. *A. vinelandii* was grown at 30 °C in Burk's nitrogen-free salts (Kennedy *et al.*, 1986), supplemented with either sucrose at 2 % (BS medium), or 0.2 % *n*-butanol (BB encystment medium). *E. coli* DH5*α* was grown on Luria–Bertani (LB) medium (Miller, 1972) at 37 °C. Antibiotic concentrations used for *A. vinelandii* and *E. coli*, respectively, were as follows: ampicillin, not used and 200 μg ml^−1^; gentamicin, 1.5 and 10 μg ml^−1^; kanamycin 6 μg ml^−1^ and not used; tetracycline, 15 and 20 μg ml^−1^. Triparental matings were carried out as reported by Kennedy *et al.* (1986). *A. vinelandii* transformation was carried out as described by Page & von Tigerstrom (1978), as modified by Bali *et al.* (1992).

### DNA techniques.

DNA isolation, cloning, Southern blotting, and nick translation procedures were carried out as described by Sambrook *et al.* (1989).

### Cloning of *flhDC, fleQ*, *mucA* and *cydR* genes.

The *A. vinelandii fleQ* and *flhDC* genes were amplified by PCR using ATCC 9046 chromosomal DNA as a template and the following oligonucleotides: *fleQ*, upper primer 5′-TTATGCCTTGCTGGGGTTGC-3; *fleQ*, lower primer 5′-TTCACCCGTTCGTAGGCATC-3′; *flhDC*, upper primer 5′-AATGCTTCCCAGGCGAGATC-3′; and *flhDC*, lower primer 5′-GACAACGATGAGACC CAGAG-3′. For *mucA* and *cydR*, oligonucleotides mucA-1U 5′-GAAATCGAGGCCACTGTG-3′, mucA-1L 5′-CAACCAATTCTGCGCATC-3′, cydRf 5′-GTTCGTTCGATCTGCATGC-3′ and cydRr 5′-TTACTGGAAGCGGACATGCG-3′ were used. Primers were designed on the basis of the OP strain draft genome sequence available at http://img.jgi.doe.gov/cgi-bin/pub/main.cgi. The resulting 2157 (*fleQ*), 1667 (*flhDC*), 910 (*mucA*) and 1173 (*cydR*) bp PCR products were cloned in pMOS*Blue*, producing plasmids pLRQ, pLRDC, pMUC and pMCYDR, respectively (Table 1). Restriction mapping and partial sequencing confirmed the identity of the inserts (data not shown).

### Construction of *fleQ* : : Gm, *flhC* : : Tc, *mucA* : : Km and *cydR* : : Gm mutations.

Plasmid pLRQ was digested with *Xho*I to eliminate a 230 bp *Xho*I internal *fleQ* fragment. The 0.8 kb *Xho*I fragment containing a gentamicin-resistance cassette isolated from plasmid pBSL141 (Alexeyev *et al.*, 1995) was ligated into the pLRQ *Xho*I-digested plasmid. Plasmid pLRQ30, containing the *fleQ* : : Gm mutation was isolated. For inactivation of the *flhC* gene, a 2.0 kb *Sma*I fragment, containing a tetracycline-resistance cassette isolated from plasmid pHP4 Ω-Tc (Fellay *et al.*, 1987), was inserted into the *Stu*I site within gene *flhC* in plasmid pLRDC. Plasmid pLRDC50 containing the *flhC* : : Tc insertion was isolated. A kanamycin-resistance cassette from plasmid pBSL99 (Alexeyev *et al.*, 1995) was introduced into the *Xho*I site of *mucA* in plasmid pMUC, producing plasmid pSRA4. For inactivation of *cydR*, a 3.0 kb *Cla*I fragment, containing a gentamicin-resistance cassette from plasmid pMS40 (Peralta-Gíl *et al.*, 2002), was introduced into the *Cla*I site present within *cydR* in pMCYDR, producing plasmid pMCYDR-Gm

Plasmids pLRQ30 and pLRDC50, which are unable to replicate in *A. vinelandii*, were used to introduce the *fleQ* : : Tc and *flhC* : : Gm mutations into strain ATCC 9046. Transformants were selected using the corresponding antibiotic, and confirmed by Southern blot analysis to carry the desired mutations (data not shown). Plasmid pSRA4 was transformed into strain ATCC 9046 to generate strain SRA4. The presence of the *mucA* : : Km mutation in strain SRA4 was confirmed by PCR analysis. Plasmid pMCYDR-Gm was introduced into strains ATCC 9046 and SRA4. A gentamicin-resistant transformant derived from ATCC 9046 was isolated, and named ATCR. No gentamicin transformants derived from strain SRA4 were obtained in three different experiments. The *cydR* : : Gm gene replacement in ATCR was confirmed by PCR analysis (data not shown).

### Construction of plasmid pLRGm-DC.

Plasmid pJB3Km1 (Blatny *et al.*, 1997), which was able to replicate in *A. vinelandii,* was digested with *Hin*dIII and *Bam*HI restriction enzymes to remove a 1 kb fragment that included the kanamycin-resistance gene. This fragment was replaced by a 1.6 kb *Bam*HI–*Hin*dIII fragment containing the *flhDC* genes, including their promoter sequences. A 0.8 kb *Bam*HI fragment encoding the gentamicin-resistance gene was introduced into the plasmid to produce plasmid pLRGm-DC, which was transferred by conjugation into strain AC30 in a triparental mating using plasmid pRK2013.

### Motility assay.

To test the motility of *A. vinelandii*, bacterial strains were grown on BS medium at 30 °C until exponential phase. Samples of the cells were then transferred to BS or BB encystment medium, containing 0.3 % agar. These plates were incubated at 30 °C for 36 or 48 h.

### Electron microscopy.

Transmission electron microscopy to visualize flagella was carried out as previously reported (Gaona *et al.*, 2004)

### Quantitative RT-PCR (qRT-PCR).

qRT-PCR was performed as reported (Noguez *et al.*, 2008). For RNA extraction, the cultures were grown in BS liquid medium. Cells were collected at the exponential phase of growth for *flhC*, and at 37 h for *cydR*. The sequences of the primers used for the qRT-PCR assays were: for *cydR*, 5′-GGC TGTCGAGACCGTATCC-3′ and 5′-ATTCGACGGGATTGAGAATG-3′; for *flhDC*, 5′-GAACATCCATTCCTCGCTGT-3′ and 5′-ATAGAGCCGGAAAGCCTTGA-3′.

## RESULTS

### AlgU activity causes loss of flagella in *A.vinelandii*

**Fig. 1. f1:**
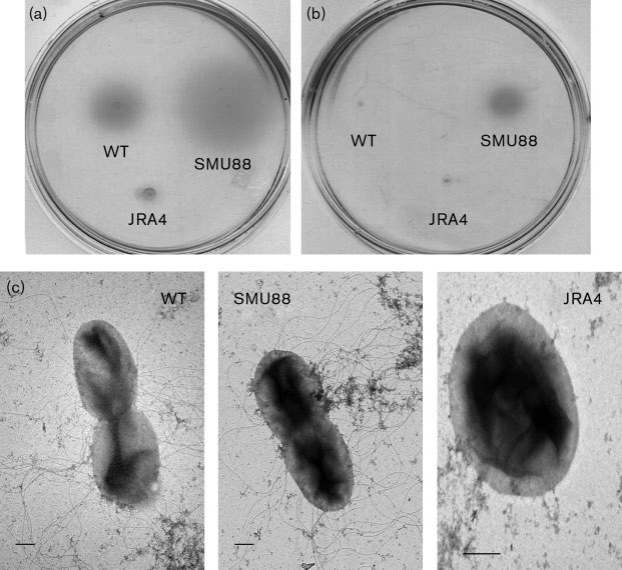
Motility phenotype of *algU* and *mucA* mutants. Swimming assays of *A. vinelandii* wild-type strain ATCC 9046 (WT), *algU* mutant SMU88, and *mucA* mutant JRA4, carried out on motility agar plates with BS medium (a) and BB encystment medium (b). (c) Electron micrographs of negatively stained preparations of strains ATCC 9046, SMU88 and JRA4. Bars, 1.0 μm. Cells for transmission electron microscopy were harvested from cultures growing exponentially on BS medium.

In *P. aeruginosa*, AlgU has a negative effect on transcription of the flagella regulon by downregulating *fleQ*. To determine whether AlgU affected flagella synthesis in *A. vinelandii*, we performed motility assays in BS medium for strains ATCC 9046, SMU88 (*algU* mutant derivative of ATCC 9046) and JRA4, a derivative carrying a *mucA* mutation that results in high AlgU activity (Núñez *et al.*, 2000). As shown in Fig. 1(a), the *mucA* mutant strain was non-motile, as indicated by the absence of a motility zone. In contrast, the *algU* mutant produced a motility zone larger than that produced by the wild-type strain. When observed under the light microscope, cells of the wild-type strain ATCC 9046 growing on BB encystment medium were non-motile. Therefore, swimming assays were also performed in BB encystment medium for the wild-type, and the *mucA* and *algU* mutants (Fig. 1b[Fig f1]). No swimming was observed for the wild-type and *mucA* strains, whereas the *algU* mutant produced a swimming zone. Using transmission electron microscopy, we examined the *mucA* and *algU* strains harvested from cultures growing exponentially on BS medium to determine the presence of flagella. In agreement with the swimming phenotype, no flagella were present for the *mucA* strain, whereas the *algU* mutant, similar to the wild-type, produced numerous flagella (Fig. 1c[Fig f1]).

### Flagella and motility genes found in the *A. vinelandii* genome

The flagella gene system of *E. coli* is one of the best studied, and is composed of over 50 genes for flagella assembly and function (Kutsukake & Nambu, 2000; Macnab, 1996). In order to identify *A. vinelandii* genes involved in flagella biogenesis and motility, and the possible targets for the AlgU-negative effect, we carried out an *in silico* analysis to search the draft genome sequence of *A. vinelandii* OP for genes homologous to bacterial flagella and motility genes. The *A. vinelandii* genome sequence data were obtained from http://img.jgi.doe.gov/cgi-bin/pub/main.cgi. Putative flagella and motility genes were identified by tblastn searches using *E. coli* genes. Because of the close phylogenetic relationship between *Azotobacter* and *Pseudomonas* species (Rediers *et al.*, 2004), we also used *P. aeruginosa* genes in the analysis.

**Fig. 2. f2:**
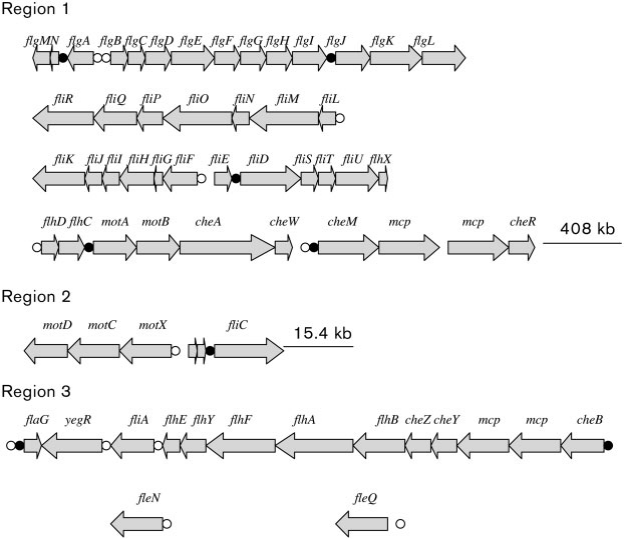
Schematic representation of the *A. vinelandii* flagellar and motility regulon. Filled and open circles represent the presence of FliA- and RpoD-recognized consensus sequences, respectively. Genes in region 1 are contiguous. In region 2, *motD* is located 408 kb downstream of *cheR*. The *fliC* gene in region 2 is separated from *flaG* by 15.3 kb. *fleQ* and *fleN* are not linked to flagella genes.

Three regions containing putative flagella and motility genes were identified in the *A. vinelandii* genome. These genes and the putative proteins they encode are listed in supplementary Table S1 (available with the online version of this paper). The genes include homologues of the *flhDC* master regulators in *E. coli*, and the *Pseudomonas* master regulator *fleQ* and its anti-activator *fleN*. In contrast to *Pseudomonas* spp., where these genes are present in the neighbourhoods of other flagella genes, the *A. vinelandii fleQ* and *fleN* genes are not linked to flagella genes. A representation of the regions, as well as the position and orientation of these genes in the *A. vinelandii* genome, is presented in Fig. 2. Region 1 consists of 39 717 bp, and contains 42 genes. Region 2 harbours four genes, including *fliC*, and region 3 consists of 12 genes.

A blast search of *A. vinelandii* FlhDC proteins revealed the absence of homologues in *Pseudomonas* spp. A summary of flagella genes present or absent in *A. vinelandii*, *P. aeruginosa* and *E. coli* is presented in supplementary Table S2 (available with the online version of this paper). Most *A. vinelandii* flagella genes showed the highest identity to genes from *Chromohalobacter salexigens* and *Cupriavidus necator* (Table S1). *C. salexigens* is a gamma proteobacterium that is closely related to *Pseudomonas* spp. and *E. coli*. Motility in *Chromohalobacter* occurs by means of peritrichous flagella (Holt *et al.*, 1994).

We also carried out a search for AlgU-, RpoD- and FliA-recognized consensus sequences within intergenic sequences of flagella genes larger than 80 nt. For putative RpoD (sigma 70)-recognized promoters, we used bprom (http://www.softberry.com/berry.phtml), which is a program for the prediction of bacterial RpoD promoters that has an accuracy of about 80 %. The search for putative FliA and AlgU promoters was carried out by ocular inspection. The results are presented in supplementary Table S3 (available with the online version of this paper) and Fig. 2. The AlgU GAACTT-16/17 bp-TCTgA-recognized sequence (Gaona *et al.*, 2004) was not identified. The FliA-recognized sequence CTAA-15 bp-GCCGATAG was found upstream of eight putative operons. Twelve genes, including the master operon *flhDC*, were found to possess putative RpoD promoters The putative *cheM–mcp–mcp–cheR* operon, and the *flaG* gene, were found to possess both FliA and RpoD consensus sequences.

### Inactivation of the *flhDC* genes, but not *fleQ,* impairs motility

**Fig. 3. f3:**
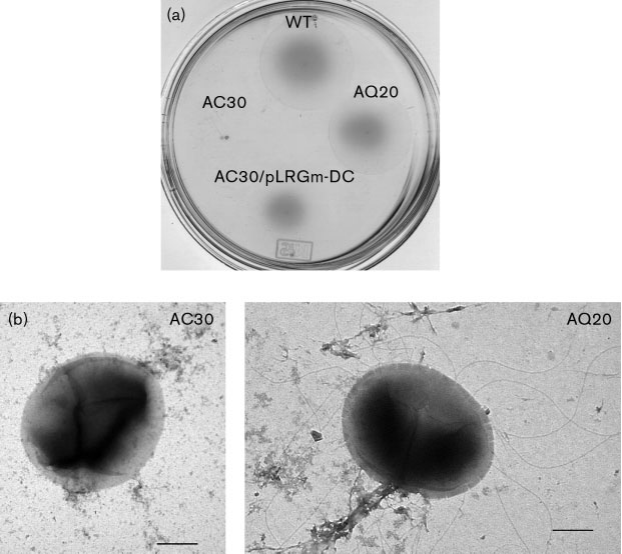
Motility phenotype of *flhC* and *fleQ* mutants. (a) Swimming plate assay carried out in BS medium for: ATCC 9046 (WT), *flhC* mutant AC30, *fleQ* mutant AQ20, and AC30/pLRGm-DC. (b) Electron micrographs of AC30 and AQ20 mutants. Bars, 1.0 μm.

In contrast to *E. coli* and *Pseudomonas* spp., *A. vinelandii* was found to possess *flhDC* and *fleQ* regulatory genes. To determine the functionality of the *flhDC* and *fleQ* homologues, which are located in the top hierarchy of flagella gene regulation in *E. coli* and *P. aeruginosa*, respectively, we constructed, as described in Methods, strain AC30 carrying a *flhC* : : *Tc* mutation, and strain AQ20 carrying a *fleQ* : : Gm mutation. The AC30 and AQ20 mutants were tested for their swimming phenotype. As shown in Fig. 3(a), inactivation of *flhC* completely inhibited motility. In contrast, the mutant carrying the *fleQ* mutation showed a motility phenotype similar to the wild-type strain. Electron microscopy revealed the absence of flagella in the *flhC* mutant, but not in the *fleQ* mutant (Fig. 3b[Fig f3]). These results indicate that *flhDC* positively controls flagella synthesis and motility in *A. vinelandii*.

### Motility is restored by complementation with the *flhDC* genes

The *flhD* and *flhC* genes overlap by 1 nt, and they are separated from the downstream *motAB* genes by an intergenic region containing a consensus FliA-recognized sequence (Fig. 2, Supplementary Table S3). Thus, the *flhDC* genes appear to constitute a bicistronic operon, and the *flhC* mutation was not expected to affect *motAB* transcription. To confirm that the swimming defect in strain AC30 was caused by the lack of the FlhC protein, and not by polar effects on downstream genes, and to confirm functionality of the *flhDC* genes, plasmid pLRGm-DC, containing only the *flhDC* genes including the promoter sequences, was introduced into strain AC30 by conjugation. The resultant strain AC30/pLRGm-DC showed a swimming phenotype similar to that of the wild-type strain (Fig. 3a[Fig f3]).

### Effect of AlgU on expression of *flhDC*

**Fig. 4. f4:**
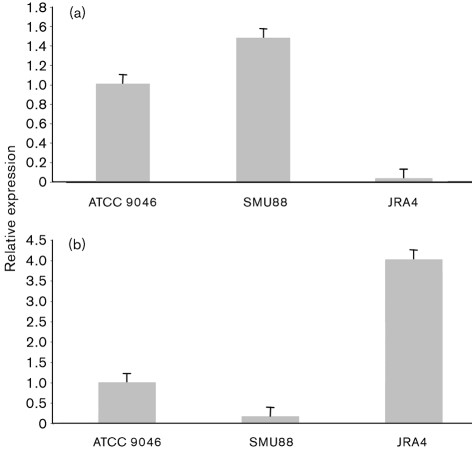
Effect of AlgU on expression of *flhDC* and *cydR*. qRT-PCR analysis of *flhDC* (a), and *cydR* (b) gene expression from BS cultures of the wild-type strain ATCC 9046, the *algU* mutant SMU88 and the *mucA* mutant JRA4. The level of *flhDC* and *cydR* transcripts was normalized according to the levels of *gyrA* mRNA, and data are presented as -fold changes of mRNA levels of SMU88 and JRA4 mutant strains relative to those of the wild-type ATCC 9046.

To determine whether AlgU affected flagella synthesis by downregulation of the master operon *flhDC*, we carried out qRT-PCR analysis to quantify the levels of *flhDC* mRNA in cells of mutant SMU88 lacking AlgU activity, and in the *mucA* mutant JRA4, in which the absence of the anti-AlgU protein MucA results in high AlgU activity (Núñez *et al.*, 2000) (Fig. 4). RNA was isolated from cultures of the *A. vinelandii* strains grown exponentially on BS medium. In the SMU88 *algU* mutant strain, *flhDC* mRNA was 40 % higher than in the wild-type. In contrast, *flhDC* mRNA levels were very low in the non-motile *mucA* strain JRA4. Based on these results, we conclude that the expression of the master *flhDC* operon is under the negative control of AlgU, although this control might not be direct.

### CydR is under AlgU control, and is likely to be a repressor of *flhDC* expression

**Fig. 5. f5:**
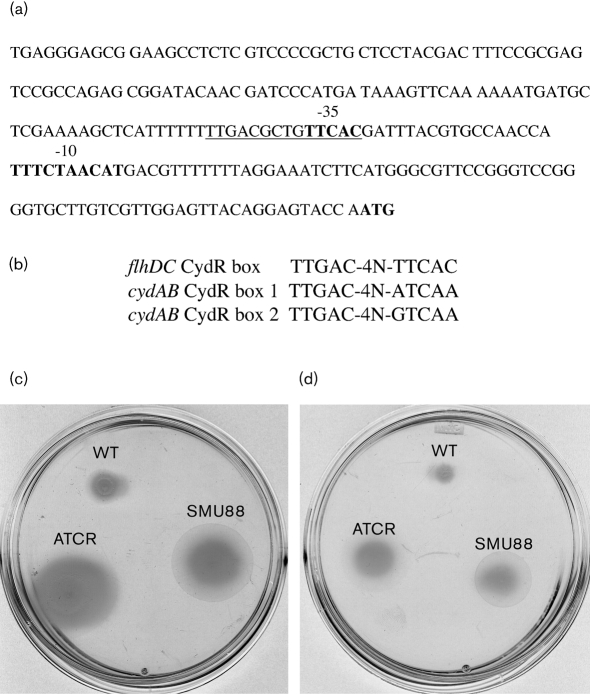
Motility phenotype of *cydR* mutant. (a) DNA sequence of the 5′ region of *flhDC*. The putative CydR-binding site is underlined. The –10 and –35 regions are shown in bold. (b) Sequence alignment of the CydR boxes. (c, d) Swimming plate assay carried out in BS medium (c), and BB encystment induction medium (d), for ATCC 9046 (WT), and the *cydR* (ATCR), and *algU* (SMU88) mutants.

We inspected the 228 nt *flhDC* promoter region for the presence of putative binding sites for known regulators. *A. vinelandii* CydR is an Fnr homologue that represses transcription of the oxidase genes *cydAB* by binding at the CydR boxes located in the *cydAB* promoter region (Wu *et al.*, 2000). A sequence highly similar to the CydR boxes is present in the *flhDC* promoter region, and it overlaps the putative –35 sequence (Fig. 5). The presence of a putative CydR box within the *flhDC* putative promoter led us to hypothesize that CydR might mediate repression of *flhDC* by AlgU. In order to determine whether *cydR* transcription was dependent on AlgU, we carried out qRT-PCR analysis to determine the levels of *cydR* mRNA in cells of the wild-type, the *algU* mutant SMU88, and the *mucA* mutant JR4. RNA was isolated from cultures of the *A. vinelandii* strains grown on BS medium. As shown in Fig. 4(b), in the *algU* mutant strain, the *cydR* mRNA level is significantly reduced compared to the wild-type, whereas the levels in the *mucA* mutant are threefold higher, indicating that AlgU is indeed required for CydR expression. Based on this result, and on the presence of CydR boxes in the *flhD* promoter, inactivation of *cydR* was expected to produce a hyper-motility phenotype similar to that of the *algU* mutant, and to restore the motility phenotype in the *mucA* mutant. We constructed, as described in Methods, strain ATCR, which is an ATCC 9046 derivative carrying a *cydR* mutation. Strain ATCR grew very poorly on Burk's medium (data not shown), but produced a motility zone larger than that produced by the *algU* mutant (Fig. 5c[Fig f5]). The swimming of ATCR on BB encystment medium was similar to that of the *algU* mutant (Fig. 5d[Fig f5]). Efforts to construct a *mucA–cydR* double mutant strain were unsuccessful, probably because of the detrimental effects on growth caused by the *cydR* mutation, and also because of the effects of the *mucA* mutation, which reduces the growth rate due to alginate overproduction (Núñez *et al.*, 2000). Taken together, these results indicate that AlgU exerts a positive control on CydR, and that this in turn is a repressor of *flhD* expression.

## DISCUSSION

*A. vinelandii* undergoes differentiation to form a metabolically dormant cyst resistant to desiccation. A mature cyst consists of a contracted cell known as the central body, which is surrounded by a capsule containing a high proportion of alginate (Page & Sadoff, 1975). Encystment can be induced in laboratory conditions by transferring vegetative motile cells grown in liquid BS medium to Burk's medium supplemented with 0.2 % *n*-butanol or *β*-hydroxybutyrate as the sole carbon source. This induction results in loss of flagella (Sadoff, 1975).

The alternative sigma factor AlgU is required for expression of the alginate biosynthesis genes in *A. vinelandii* (Gaona *et al.*, 2004; Moreno *et al.*, 1998). Alginate is essential for the formation of mature cysts, and mutations in the alginate biosynthesis genes, or in *algU*, impair alginate synthesis and encystment (Campos *et al.*, 1996; Mejía-Ruíz *et al.*, 1997; Moreno *et al.*, 1998). A link between alginate synthesis and flagellum expression, which are inversely regulated by the alternative sigma factor AlgU, has been shown in *P. aeruginosa* (Tart *et al.*, 2005, 2006), which is a close relative of *A. vinelandii*.

We have shown here that loss of motility upon encystment induction in *A*. *vinelandii* is caused by AlgU activity. Thus, as in the case of *P. aeruginosa,* alginate synthesis and flagella biogenesis, are inversely controlled by AlgU. In order to identify possible targets for AlgU among the flagella genes, we identified the *A. vinelandii* gene homologues of bacterial flagella and motility genes. Most of these genes share the highest identity with the genes of *C. salexigens*; this bacterium is phylogenetically closely related to *A. vinelandii*, since their 16S rRNA shares 90.86 % similarity, and this level of identity is second only to *Pseudomonas* spp., for which the similarity is around 95–96 %.

An important finding of this study was the presence in *A. vinelandii* of *fleQ* and *flhDC*, which are the master regulators of flagella biogenesis. *flhDC*, but not *fleQ*, is located in the context of other flagella genes. Inactivation of the *flhDC* and *fleQ* genes indicated that the FlhDC proteins are the master regulators of flagella biogenesis in *A. vinelandii*.

The lack of FleQ involvement in flagella biogenesis is in agreement with the absence of the *fleRS* genes (Table S2), which are the targets of FleQ regulation in *P. aeruginosa* (Dasgupta *et al.*, 2003). It is possible that, in *A. vinelandii*, FleQ participates in regulating the expression of other genes that are not involved in flagella biogenesis.

This study showed that in *A. vinelandii*, AlgU activity inhibits flagella synthesis in vegetative cells and under encysting conditions. A negative effect of AlgU on transcription of the *flhDC* operon was also shown here. In *P. aeruginosa*, negative regulation of flagella synthesis by AlgU is carried out by activating expression of the transcriptional regulator AmrZ, which in turn represses *fleQ* transcription (Tart *et al.*, 2005, 2006). Interestingly, binding sites for CydR (CydR boxes) within the *flhDC* promoter region were identified, suggesting that CydR is a repressor of *flhDC* expression. In agreement with this proposal, inactivation of *cydR* conferred a hyper-swimming capacity to the wild-type strain. In addition, we showed that the levels of *cydR* mRNA are significantly reduced in the *algU* mutant, indicating that AlgU is required for *cydR* expression, and that CydR mediates the negative effect of AlgU. Inactivation of *cydR* was therefore expected to restore motility in the *mucA* mutant. Unfortunately, our efforts to isolate a *mucA–cydR* double mutant were unsuccessful.

**Fig. 6. f6:**
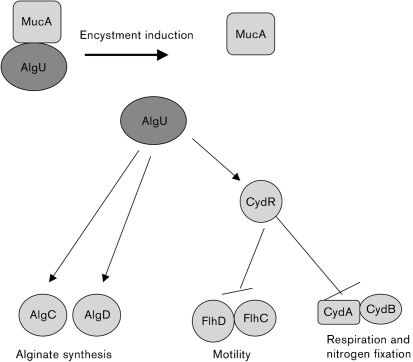
Model for the control by AlgU of: motility, alginate synthesis, respiration, and nitrogen fixation upon encystment induction in *A. vinelandii*. Induction of encystment, which is likely to be caused by a stress condition, results in the release of AlgU by MucA. AlgU in turn activates transcription of alginate biosynthesis genes and CydR, which is the repressor of FlhDC and CydAB.

CydR is a homologue of Fnr. In *A. vinelandii*, CydR has been shown to act as a repressor of the *cydAB* genes encoding cytochrome bd, which is required for aerotolerant nitrogen fixation (Wu *et al.*, 2000). The loss of flagella and nitrogen fixation activity observed upon induction of encystment (Sadoff, 1975; Hitchins & Sadoff, 1973) can now be explained by the negative effect of CydR on expression of the *cydAB* and *flhDC* genes. Taken together, the results presented in this study indicate the existence of this regulatory cascade consisting of AlgU upstream of CydR, which in turn acts as a repressor of *flhCD* expression. A model for the control of alginate synthesis, motility, respiration and nitrogen fixation, upon encystment induction by the AlgU–CydR regulatory cascade in *A. vinelandii*, is shown in Fig. 6.

This study also showed that the common feature in the regulation of motility in both *P. aeruginosa* and *A. vinelandii* is the negative effect of AlgU, but that there is a difference in the repressor controlled by AlgU (AmrZ for *Pseudomonas*, and CydR for *Azotobacter*), and the targets of these repressors (*fleQ* for *Pseudomonas*, and *flhDC* for *A. vinelandii*). These differences may be explained by the need of *A. vinelandii* to coordinate loss of functions such as motility, high respiration rate and nitrogen fixation that occur upon encystment induction, which is a process not carried out by *Pseudomonas* species.

## Figures and Tables

**Table 1. t1:** Bacterial strains and plasmids used in this work

**Strain or plasmid**	**Relevant characteristics**	**Source or reference**
***A. vinelandii***		
ATCC 9046	Wild-type	ATCC
SMU88	ATCC 9046 with an *algU* : : Km mutation	Moreno *et al.* (1998)
JRA4	ATCC 9046 with *mucA* : : Gm mutation	Núñez *et al.* (2000)
SRA4	ATCC 9046 with a *mucA* : : Km mutation	This work
AQ 20	ATCC 9046 with a *fleQ* : : Gm mutation	This work
AC 30	ATCC 9046 with a *flhC* : : Tc mutation	This work
ATCR	ATCC 9046 with a *cydR* : : Gm mutation	This work
***E. coli***		
DH5*α*	*supE44lacU169hsdR17recA1endA1gyrA96thi1relA1*	Gibco-BRL
**Plasmids**		
pMOS*Blue*	Plasmid used for cloning PCR products	Amersham
pLRQ	pMOS*Blue* derivative carrying a 2.1 kb DNA fragment containing *A. vinelandii fleQ* gene amplified by PCR	This work
pLRDC	pMOS*Blue* derivative carrying a 1.6 kb DNA fragment containing *A. vinelandii flhCD* genes amplified by PCR	This work
pLRQ30	pLRQ derivative containing a *fleQ* : : Gm mutation	This work
pLRDC50	pLRDC derivative containing a *flhC* : : Tc mutation	This work
pMUC	pMOS*Blue* derivative carrying a 0.91 kb DNA fragment containing *A. vinelandii mucA* gene amplified by PCR	This work
pSRA4	pMUC derivative containing a *mucA* : : Km mutation	This work
pMCYDR	pMOS*Blue* derivative carrying a 1.17 kb DNA fragment containing *A. vinelandii cydR* gene amplified by PCR	This work
pMCYDR-Gm	pCYDR derivative containing a *cydR* : : Gm mutation	This work
pJB3Km1		Blatny *et al.* (1997)
pRK2013		Hedges & Baumberg (1973)
pHP4 Ω-Tc	Source of the Tc^r^ cassette	Fellay *et al.* (1987)
pBSL141	Source of the Gm^r^ cassette	Alexeyev *et al.* (1995)
pBSL99	Source of the Km^r^ cassette	Alexeyev *et al.* (1995)
pSM40	Source of the Gm^r^ cassette	Peralta-Gíl *et al.* (2002)
pLRGm-DC	pJB3Km1 derivative carrying a 1.6 kb DNA fragment containing *A. vinelandii flhCD* genes and the Gm^rh^ gene from pBSL14	This work

## Abbreviations

- qRT-PCR, quantitative RT-PCR.
